# Perirenal hemorrhage associated with feline infectious peritonitis: a novel presentation of a classic disease

**DOI:** 10.1177/10406387251341239

**Published:** 2025-05-29

**Authors:** Madeleine I. Gauthier, Carolyn Legge, Dayna A. Goldsmith, Maria Bravo Araya, Jennifer L. Davies

**Affiliations:** Faculty of Veterinary Medicine, University of Calgary, Calgary, Alberta, Canada

**Keywords:** feline, feline coronavirus, feline infectious peritonitis, immunohistochemistry, perirenal hemorrhage

## Abstract

Feline infectious peritonitis (FIP), caused by a mutated biotype of feline coronavirus (FCoV; *Coronaviridae*, *Alphacoronavirus*), is a significant disease of felids. We investigated perirenal hemorrhage, an unreported lesion in FIP, through a retrospective analysis of 51 immunohistochemistry-confirmed FIP cases submitted to the Diagnostic Services Unit (DSU; University of Calgary, Calgary, Alberta, Canada) between 2010 June 30 and 2024 June 30. Five cats had perirenal hemorrhage in the right retroperitoneal space; 4 had concurrent subcapsular renal hemorrhage; and 1 had sublumbar muscle hemorrhage and hemoabdomen. One case had additional hemorrhages in the brain and cervical spinal cord. Concurrent gross lesions typical of FIP included pyogranulomatous inflammation in various organs and protein-rich cavitary effusions. Histologic lesions typical of FIP (vasculitis and pyogranulomatous inflammation) were present in the kidneys and retroperitoneal fat of 4 cases, and in 3 cases, FCoV antigen was demonstrated in the regions of hemorrhage. The exact mechanism of this hemorrhage is unknown, but we speculate that vasculitis caused by FIP is the cause. Despite the relatively low prevalence of perirenal hemorrhage in this cohort, this lesion represents a unique, previously unreported manifestation of FIP that clinicians and pathologists should be aware of and consider in the differential diagnosis for fluid accumulation or space-occupying lesions in the retroperitoneum of cats.

Feline infectious peritonitis (FIP) is one of the most important infectious diseases of cats, and nearly every small animal practitioner and veterinary pathologist will encounter cases during their careers.^7,8,12,14^ It is an immune-mediated disease caused by a mutated biotype of feline coronavirus (FCoV; *Coronaviridae*, *Alphacoronavirus*).^16,17^ On autopsy, the most prevalent finding is fibrinous serositis, manifesting mainly as peritoneal exudative effusion, with lesser involvement of the pleura, pericardium, and scrotum (effusive form).^9,12^ Other common findings include pyogranulomas on serosal surfaces, extending into multiple organs along the vasculature, with frequent enlargement of thoracic and abdominal lymph nodes (dry form). Within the CNS, FIP may cause meningoencephalitis and myelitis. Ocularly, it may cause unilateral or bilateral uveitis and chorioretinitis. Histologically, FIP causes generalized vasculitis and perivasculitis, especially phlebitis, with pyogranulomatous inflammation tracking the vasculature. The gold standard for diagnosing FIP is immunohistochemistry (IHC) to detect FCoV antigen within macrophages in tissues with characteristic histologic lesions of FIP; IHC has 97–100% sensitivity and specificity of up to 100%.^5^ Due to the invasive and high-risk nature of antemortem tissue collection, FIP is typically diagnosed at autopsy.

Anatomic pathologists at the Diagnostic Services Unit (DSU; University of Calgary Faculty of Veterinary Medicine [UCVM], Calgary, Alberta, Canada) began observing cases of FIP with perirenal hemorrhage in 2014. To date, this lesion is not described in the literature, to our knowledge, and we consider it novel. The purpose of our retrospective study is to describe this novel FIP lesion and its prevalence among cats diagnosed with FIP at the DSU since the laboratory’s inception in 2010.

We queried the DSU Laboratory Information Management System (LIMS; Vetstar Animal Disease Diagnostic System [VADDS], Advanced Technology) for all cases of FIP accessioned between 2010 June 30 and 2024 June 30. We searched the diagnostic codes “feline infectious peritonitis”, “interstitial nephritis”, “nephritis”, “renal hemorrhage”, and “vasculitis”, and additionally completed a free text search for “feline infectious peritonitis”. We retrospectively reviewed case reports of all whole-body and field autopsies submitted to the DSU with gross and/or histologic lesions consistent with FIP during this period. The data we obtained from each case included age, sex, breed, cause of death, method of diagnosis, descriptions of gross and histologic lesions, and presence or absence of perirenal hemorrhage. We included only cases with histologic lesions (perivasculitis, vasculitis, and pyogranulomatous inflammation) compatible with FIP and verified by FCoV IHC. We reviewed archived H&E-stained sections from cases that were grossly and/or histologically diagnosed with FIP but did not have IHC confirmation to verify the presence of pyogranulomatous inflammation and vasculitis in at least one organ; the corresponding formalin-fixed, paraffin-embedded tissue blocks were sent for IHC.

Immunohistochemical staining was conducted at Prairie Diagnostic Services (Saskatoon, SK, Canada) using an automated slide stainer (Autostainer Plus; Agilent). Epitope retrieval was performed in a Tris/EDTA pH 9 buffer at 97ºC for 20 min. The primary antibody, mouse anti-FIP clone FIPV3-70 (Custom Monoclonals), was used at a 1:1,000 dilution and applied for 30 min at room temperature. Binding of the primary antibody was detected using a horseradish peroxidase–labeled polymer detection reagent (EnVision+ System-HRP labelled polymer; Agilent). The staining was visualized using 3,3′-diaminobenzidine tetrahydrochloride (Dako liquid DAB+ substrate chromogen system; Agilent) as the chromogen, and counterstained with Mayer hematoxylin.

We identified 61 cases of FIP diagnosed at the DSU between 2010 June 30 and 2024 June 30. Of these, 34 had previous positive IHC results, and 26 cases were sent for confirmatory IHC testing; 17 of 26 were positive. Histopathology was not pursued in one case and thus was excluded from our study. We included 51 cases of FIP that met our inclusion criteria of compatible histologic lesions and IHC positivity. Of those cases, 43 were full-body autopsies, 5 were field autopsies, and 3 were surgical biopsies. The median age of affected cats was 9 mo, and the mean age was 18 mo (range: 3–120 mo). The cohort was comprised of 28 males (19 castrated) and 23 females (12 spayed). The population consisted of 24 domestic shorthair cats, 5 domestic longhair cats, 3 Siberians, 2 British Shorthair cats, 2 domestic medium hair cats, 2 Maine Coons, 2 Orientals, 2 Ragdolls, 1 Bengal, 1 Cornish Rex, 1 LaPerm, 1 Persian-Himalayan X, 1 Savannah, 1 Scottish Fold, and 3 non-specified breeds. Of the cats that died, 37 were euthanized and 8 died naturally. In 3 cases, the history did not specify whether the cats died naturally or were euthanized.

At autopsy, fibrinous serositis was noted in 32 of 51 cases, characterized by at least one of peritoneal (30), pleural (19), pericardial (12), or scrotal (1) effusion with fibrin strands. Pyogranulomatous inflammation was present in 42 cases, seen as multiple, 0.1–2-cm, white-to-tan nodules on the serosal surface of and extending into at least one organ. The distribution of organs was as follows: mesenteric lymph nodes (22), kidney (21), liver (17), colon (9), spleen (9), small intestines (8), lung (5), brain or spinal cord (2), frontal sinuses (2), and pancreas (1). Icterus was present in 18 cases. In 9 cases, there was pallor of the mucous membranes and conjunctiva, and thin watery blood (interpreted as anemia). Microscopically, pyogranulomatous-to-lymphoplasmacytic inflammation with necrotizing vasculitis or perivasculitis was present in all 51 cases, with the following distribution of organs: liver (29), mesenteric lymph nodes (28), kidney (26), lung (26), peritoneum (25), spleen (16), brain or spinal cord (13), colon (13), small intestine (12), eye (9), pancreas (7), diaphragm (5), pleura (5), heart (4), nasal turbinates (3), pericardium (3), and testis (1).

Pathologists identified perirenal hemorrhage in 5 of 51 cases. Case 1 was a 5-mo-old, female spayed, domestic shorthair cat. Case 2 was a young adult, intact female, Cornish Rex. Case 3 was a 12-mo-old, castrated male, domestic longhair cat. Case 4 was an 8-mo-old, castrated male, domestic shorthair cat. Case 5 was a 6-mo-old, intact male, Oriental. All 5 cats had been euthanized intravenously with pentobarbital sodium. Of these cases, 2 were dehydrated and 4 were in poor nutritional condition. Icterus was present in 4 cases, and anemia was noted in 2 cases. All 5 cases had peritoneal effusion, 3 cases also had pleural effusion, and 2 cases had pericardial effusion. Grossly, pyogranulomas were evident in the kidney (4), colon (2), liver (2), mesenteric lymph nodes (2), spleen (2), lungs (1), and peritoneum (1). Histologically, pyogranulomatous-to-lymphoplasmacytic inflammation with necrotizing vasculitis/perivasculitis was present in the following organs: kidney (4), liver (3), lung (3), mesenteric lymph nodes (3), spleen (3), colon (2), small intestine (2), brain or spinal cord (1), nasal turbinates (1), pericardium (1), and pleura (1).

In all 5 cases, hematomas were observed in the right retroperitoneal space and were 2–6-cm diameter (Fig. 1A). In one case, this hemorrhage was located caudal to the right kidney, displacing and compressing the right ureter ventrally. In another case, the hemorrhage extended into the right sublumbar muscles; hemoabdomen was also present. Four of the 5 cases had subcapsular hemorrhage in the right kidney (Fig. 1B). In 3 cases, histopathology captured the perirenal hemorrhage in association with pyogranulomatous nephritis and vasculitis (Fig. 1C), and IHC demonstrated variable amounts of FCoV antigen in the lesions (Fig. 1D). In one case, the kidney section captured the pyogranulomatous nephritis and vasculitis but did not capture the perirenal hemorrhage. In this case, FCoV antigen was present in the renal lesion. In one case, there were neither gross nor histologic lesions in the kidneys to account for the perirenal hemorrhage. Multifocal hemorrhages were noted in the leptomeninges of the brain and cervical spinal cord in one case, and, on histopathology, this hemorrhage was associated with pyogranulomatous meningitis and vasculitis.

**Figure 1.**
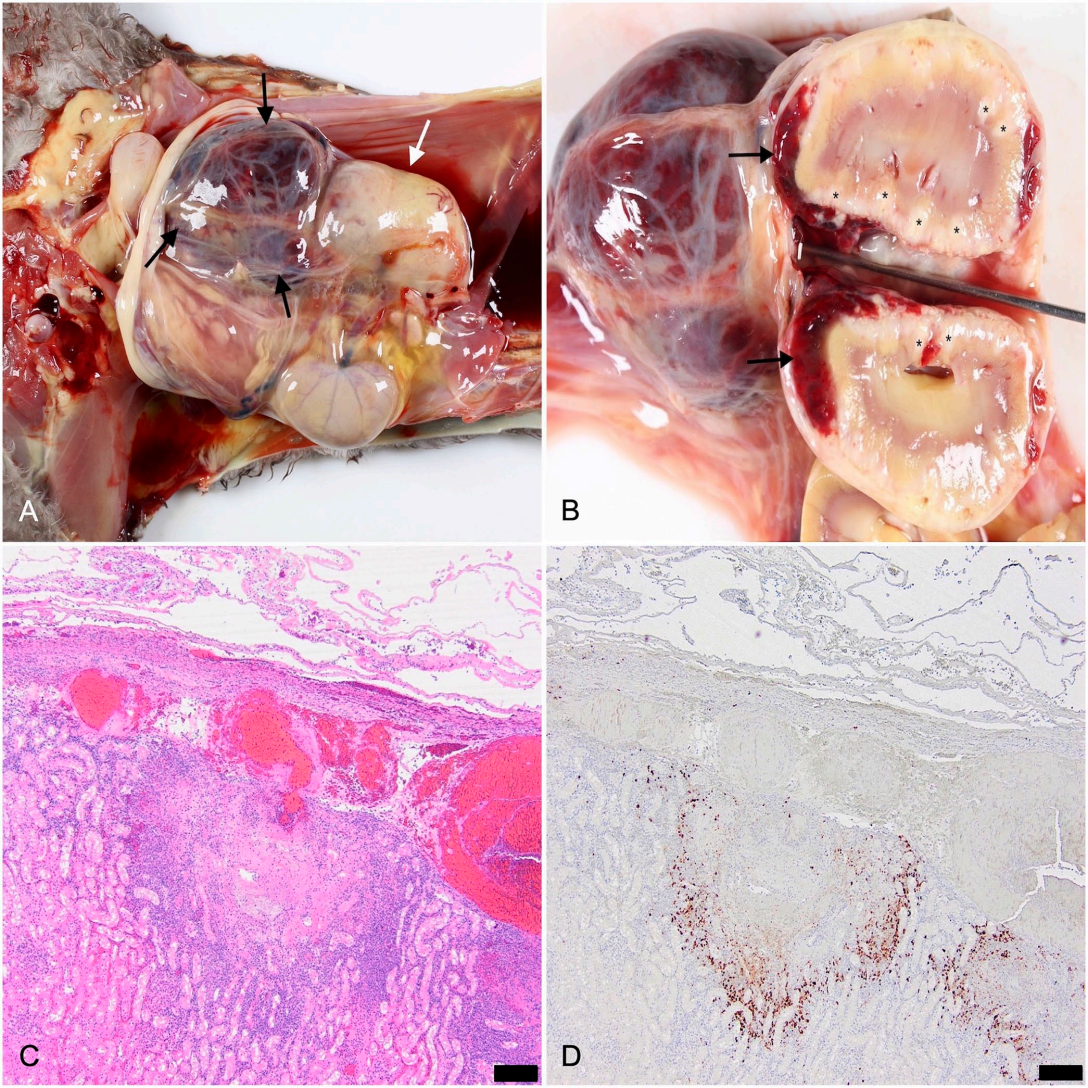
Feline infectious peritonitis with perirenal hemorrhage in case 2. **A.** Caudal to the enlarged right kidney (white arrow) lies a hematoma (black arrows) in the retroperitoneal space. **B.** Longitudinal section of the kidney with subcapsular renal hemorrhage (black arrows) and pyogranulomas tracking the vasculature (*). **C.** Multifocal pyogranulomatous nephritis, necrotizing vasculitis, and perirenal hemorrhage. H&E. Bar = 200 µm. **D.** Strong immunoreactivity to feline coronavirus antigen in a granular cytoplasmic pattern associated with the pyogranulomatous nephritis, vasculitis, and near the perirenal hemorrhage. Immunohistochemistry. Bar = 200 µm.

Retroperitoneal hemorrhage is well-described in animals; in some instances, rupture of retroperitoneal hematomas can be fatal. At this location, hemorrhage may dissect widely, making it challenging to pinpoint the origin.^15^ Reported causes of retroperitoneal hemorrhage in small animals include trauma, coagulopathies, renal and adrenal gland tumors, retroperitoneal foreign bodies or tumors, and vascular anomalies.^20^ Retroperitoneal hemorrhage in cats has also been associated with renal pelvic rupture and a chronic expanding hematoma.^2,4^

FIP has not been reported as a cause of perirenal hemorrhage in cats, to our knowledge. We retrieved no cases of perirenal hemorrhage in association with FIP in a search of Google, PubMed, CAB Direct, Web of Science, and Scopus, using the search terms “cat”, “feline infectious peritonitis”, and “perirenal hemorrhage”. In our study, 5 of 51 FIP cases diagnosed at autopsy had perirenal hemorrhage, a lesion that we regard as novel. In all 5 cases, the hemorrhage was localized to the right side of the retroperitoneum. We do not know the cause of this asymmetry, as the only notable difference between the feline kidneys is their position, with the right kidney situated more cranially.

The pathogenesis of this lesion remains unclear. Hemorrhage is unusual in FIP, except for hyphema, which results from vasculitis in the uvea.^10,14^ This suggests that a similar mechanism may account for the perirenal hemorrhage, with renal and perirenal vascular injury causing leakage of blood into the retroperitoneal space. This theory is supported by the vasculitis in the kidneys in 4 of 5 cases, the close association of the perirenal hemorrhage with FCoV antigen in 3 of 5 cases, and by the meningitis and vasculitis in the cat with brain and cervical spinal cord hemorrhage. However, one case did not have gross or microscopic renal lesions accompanying the perirenal hemorrhage, and alternative mechanisms for hemorrhage need to be considered.

In humans, spontaneous perirenal hemorrhage (SPH; Wünderlich syndrome) is a life-threatening condition defined as spontaneous, nontraumatic renal bleeding limited to the subcapsular and/or perirenal spaces.^11^ The most common causes of SPH in humans are renal neoplasia and vascular disorders; SPH is associated less frequently with inflammatory renal lesions, hematologic disorders, and anticoagulation therapy. Severe acute respiratory syndrome 2 (SARS-CoV-2 infection) has been implicated as a cause of SPH. The proposed mechanism of SPH was widespread endothelial damage caused by SARS-CoV-2 infection, leading to disseminated intravascular coagulation (DIC) and coagulopathy.^1,11,13^ Likewise, thrombotic events can occur in FIP, and high levels of D-dimers, along with other signs of DIC, can be seen in the end stages of FIP in both natural and experimental infections.^18,19^ Therefore, consumptive coagulopathy represents an alternate pathogenesis for perirenal hemorrhage in these cats. Dengue hemorrhagic fever in humans has a pathogenesis and lesion profile similar to FIP, including the development of DIC and perirenal hemorrhage, further supporting this alternative pathogenetic mechanism.^3,6,19^ However, due to incomplete historical information, we could not determine whether these cats had prolonged clotting times, elevated D-dimer levels, thrombocytopenia, or other clinical indicators of DIC.

The primary limitation of our study is its retrospective nature, leading to a lack of complete historical data, including clinical signs, bloodwork results, medical treatments, and cause of death. Critical information, such as the antemortem detection of hemorrhage, CBC and serum chemistry results, and clotting times, could not be reliably compared. Future prospective studies should investigate the antemortem visualization of perirenal hemorrhage in FIP cases and explore the association between DIC or other comorbidities and the occurrence of this lesion. Additionally, further research is justified to assess the prevalence of perirenal hemorrhage in FIP cases at other diagnostic centers and to explore genome sequencing for different strains of FCoV and their corresponding lesions. Although perirenal hemorrhage had a relatively low prevalence in FIP cases submitted to the DSU, our findings suggest that it is a unique manifestation of the disease that warrants recognition by clinicians and pathologists.
